# Brain metastases: Comparing clinical radiological differences in patients with lung and breast cancers treated with surgery^☆^

**DOI:** 10.1016/j.wnsx.2024.100391

**Published:** 2024-05-01

**Authors:** Daniele Armocida, Giuseppa Zancana, Andrea Bianconi, Fabio Cofano, Alessandro Pesce, Brandon Matteo Ascenzi, Paola Bini, Enrico Marchioni, Diego Garbossa, Alessandro Frati

**Affiliations:** a Experimental Neurosurgery Unit, IRCCS “Neuromed”, via Atinense 18, 86077, Pozzilli, IS, Italy; b Department of Neuroscience “Rita Levi Montalcini”, Neurosurgery Unit, University of Turin, Via cherasco 15, 10126, Turin, TO, Italy; c Human Neurosciences Department Neurosurgery Division “La Sapienza” University, Policlinico Umberto 6 I, viale del Policlinico 155, 00161, Rome, RM, Italy; d Neurosurgery Unit Department, Santa Maria Goretti Hospital, Via Guido Reni, 04100, Latina, LT, Italy; e Independent Neuroresearcher Member of Marie Curie Alumni Association (MCAA), Via Dante Alighieri 103, 03012, Anagni, FR, Italy; f IRCCS foundation Istituto Neurologico Nazionale Mondino, Via Mondino, 2, 27100, Pavia, Italy

**Keywords:** Brain metastases, Lung cancer, NSCLC, Breast cancer, Brain tumor

## Abstract

**Purpose:**

Brain metastases (BMs) most frequently originate from the primary tumors of the lung and breast. Survival in patients with BM can improve if they are detected early. No studies attempt to consider all potential surgical predictive factors together by including clinical, radiological variables for their recognition.

**Methods:**

The study aims to simultaneously analyze all clinical, radiologic, and surgical variables on a cohort of 314 patients with surgically-treated BMs to recognize the main features and differences between the two histotypes.

**Results:**

The two groups consisted of 179 BM patients from lung cancer (Group A) and 135 patients from breast cancer (Group B). Analysis showed that BMs from breast carcinoma are more likely to appear in younger patients, tend to occur in the infratentorial site and are frequently found in patients who have other metastases outside of the brain (46 %, p = 0.05), particularly in bones. On the other hand, BMs from lung cancer often occur simultaneously with primitive diagnosis, are more commonly cystic, and have a larger edema volume. However, no differences were found in the extent of resection, postoperative complications or the presence of decreased postoperative performance status.

**Conclusion:**

The data presented in this study reveal that while the two most prevalent forms of BM exhibit distinctions with respect to clinical onset, age, tumor location, presence of extra-cranial metastases, and lesion morphology from a strictly surgical standpoint, they are indistinguishable with regard to outcome, demonstrating comparable resection rates and a low risk of complications.

## 1 Introduction

Brain metastases (BM) as secondary neoplasms are the most common type of brain tumors in adults.^1^^,^^2^ When a primary tumor metastasizes to the brain, the patient's prognosis is significantly reduced to 1–2 months if untreated^3^^,^^4^; Overall survival (OS) can be improved for up to six months if treated with systemic therapies, surgery, or radiation, especially if they are detected and diagnosed early.^5^ Lung and breast cancers are two major cancers causing BMs,^2^^,^^3^ and there is limited research comparing the clinical and radiological differences between these two entities that can guide the best therapeutic approach.

The choice of therapy is generally guided by the number and location of BMs,^6^ the extent and prognosis of systemic disease, and the patient's performance status.^7^ Patients with minimal systemic disease, good performance status, and solitary BMs in a non-eloquent brain site are often treated with surgical resection followed by radiation therapy.^8^^,^^9^

The treatments described that have yielded better results in terms of outcome and disease control are surgery and radiosurgery.^10^ Survival with surgery has been found to be better than with radiosurgery,^11^ but related complications are greater. Even if open surgery turns out to be the preferred choice, patients often manifest different outcomes among themselves.^5^^,^^12^ More recent studies have shown that radiographic features could add clinical value to prognosticate the outcome in breast and lung cancer surgically-treated patients with BM^13^, ^14^, ^15^; however, there are no studies that attempt to consider all potential predictive factors together by including clinical, radiological, and surgical variables for their recognition. In this study we analyzed a cohort of patients with BMs from lung and breast cancer treated surgically, to seek information on the characteristics and clinical course of such patients. Thus, the study aims to analyze all clinical, radiologic, and surgical variables simultaneously to recognize the main histotype earlier and direct toward the correct treatment choice.

## 2 Methods

This is a retrospective observational multicentric study that collected a series of surgically-treated patients for BM between January 2016 and December 2020. The study was approved by our institutional review board (Rif. 6961 Prot. 0296/2023). Before the surgical procedure, all the patients gave informed written consent after appropriate information. Patients gave informed consent for the publication of data results. Data reported in the study have been completely anonymized. Obviously, no treatment randomization was carried out. The study is consistent with the Helsinki declaration of Human Rights in Medical Research.

### 2.1 Participants and eligibility

All the patients included in the final cohort meet the following inclusion criteria:

- Adult patients with a diagnosis of BM from breast cancer or NSLC candidates for surgery
- Preoperative Karnofsky performance status (KPS) scale >50 % (Including cases with symptomatic improvement potential due to tumor and edema, as well as cases with irreversible poor overall health)
- Estimated overall survival (OS) of >3 months (according to the radiation therapy oncology group and the grade prognostic assessment (GPA) rankings)^17^

The estimated target of the surgical procedure was the gross-total, near-total- or sub-total resection of the lesions; no biopsies were included. Patients with sub-centimetric heteroplastic lesions were included after dedicated conformational radiotherapy regimens. Only patients who underwent post-surgical adjuvant chemo-radiotherapy and a follow-up program were included.

All patients underwent a general medical, a neurological, and an oncological evaluation at admission. For all patients, we recorded gender, age, peri and post-operative KPS, clinical presentation, and tumor- and surgery-related variables: number, location, side of the lesions, tumor and edema volume and morphology.

The occurrence time of BM is defined as synchronous or metachronous tumors. “Synchronous” tumors refer to cases in which the second primary cancer is diagnosed within 6 months of the primary cancer; “metachronous” tumors refer to cases in which the second primary cancer is diagnosed more than 6 months after the diagnosis of the first primary cancer.^16^

The digital institutional database obtained clinical information. A particular focus was on the performance status expressed as KPS results. This score was chosen since it is considered to be critical for patient's survival when BM are present.^18^ KPS was recorded before surgery at the time of diagnosis, it was repeated at the second clinical evaluation within 3 months of the surgery and it was further recorded at the end of the adjuvant treatment 6 months after the surgical procedure.

### 2.2 Preoperative protocol for radiological evaluation

All patients received a pre-operative brain magnetic resonance imaging (MRI) scan including a 3 T volumetric study with the following sequences: T2w, Fluid Attenuated Inversion Recovery (FLAIR), Isotropic Volumetric T1-weighted Magnetization-prepared Rapid Acquisition Gradient Echo (MPRAGE) before and after intravenous administration of paramagnetic contrast agent. For each patient the hemisphere involved was reported (reporting if left, right or in case of tumors involving the midline). The brain lobe involved was recorded considering the one with the greatest presence of contrast-enhancing tissue in MRI (distinguishing tumors involving the frontal, temporal, parietal, occipital lobes and cases of sub-tentorial involvement). We have paid particular attention to reporting cases of brain metastases located in a deep site with involvement of the ventricle as strongly indicative of poor prognosis and high risk of insufficient resection or detection of post-operative deficits, identifying them as “periventricular/deep-seated ".

The volume of the contrast-enhancing lesion was calculated by drawing a region of interest (ROI) in a Volumetric enhancing post-contrast study weighted in T1 (a multi-voxel study), conforming to the margins of the contrast-enhancing lesion. The volume of edema was measured by drawing a ROI in a FLAIR weighted research, from which the previously calculated lesion was subtracted. The study was carried out using the Horos Dicom Viewer (v 3.36, opensource software, Pixmeo SARL, Bernex, Switzerland; https://horosproject.org/).^19^

Every patient included performed total-body sodium-enhanced computed tomography (CT) and bone scintigraphy to complete the oncology staging protocol. We then subsequently reported whether the patients at the time of the radiological diagnosis of BM had other intracranial and extracranial localizations not known at the first diagnosis and separately reporting the presence of lesions involving the bone.

### 2.3 Operative protocol for surgical evaluation

In a standard neurosurgical theatre, all the procedures were performed with an infrared-based Neuro-navigator (Brainlab, Kick® Purely Navigation), with a standard operative microscope. During the first post-operative day, as routine, the patients underwent a CT scan to exclude major complications and a volumetric Brain MRI scan to evaluate the extent of resection (EOR). During surgery, tumor excision was stopped when after the resection (en-bloc or piecemeal) white matter appeared disease-free in each aspect of the surgical cavity or, despite a directly visualized or a Navigation proven remnant, neuromonitoring or intraoperative neuropsychological testing outlined a risk for postoperative sensory-motor. Lesions were identified as cystic if they had a fluid-filled mass with evidence of a wall, solid if they consisted only of heteroplastic tissue at the time of excision, and hemorrhagic if they had blood clots in or around the tumor. For each patient operated on, the presence of an appropriate cleavage and resection plane was recorded, which therefore allowed the tumor to be removed as “en-bloc” without highlighting phenomena of peri-tumor infiltration. The percentage of patients for whom a surgical resection plane was identified in the study was therefore reported. A close-range dedicated neuro-imaging follow-up program was routinely performed in our Institution. This program included: a standard early (maximum 24 h after surgery) postoperative volumetric brain MRI. At approximately one month from surgery (25–35 days), a volumetric brain MRI scan was repeated for a first step follow-up control and information for the radiation treatment planning. In this phase, particular attention was paid to recording any complications directly linked to surgery such as the presence of bleeding from the surgical cavity, the presence of infections, wound closure defects and the presence of CSF leak.

A volumetric brain MRI scan was performed every 3 months at the end of irradiation. We performed a complete medical and neurological outpatient re-evaluation at every radiological reevaluation.

### 2.4 Size, statistics, and potential source of bias

The study size is given by the selection of the inclusion criteria. The sample was analyzed with SPSS v18 (SPSS Inc., Released 2009, PASW Statistics for Windows, Version 18.0, Chicago, Illinois, USA) to outline potential correlations between variables under investigation. Comparisons between nominal variables was carried out using the Chi2 test. Continuous variables correlations have been investigated with Pearson's Bivariate correlation. The threshold of statistical significance was considered p < 0.05.

## 3 Results

**Fig. 1:**
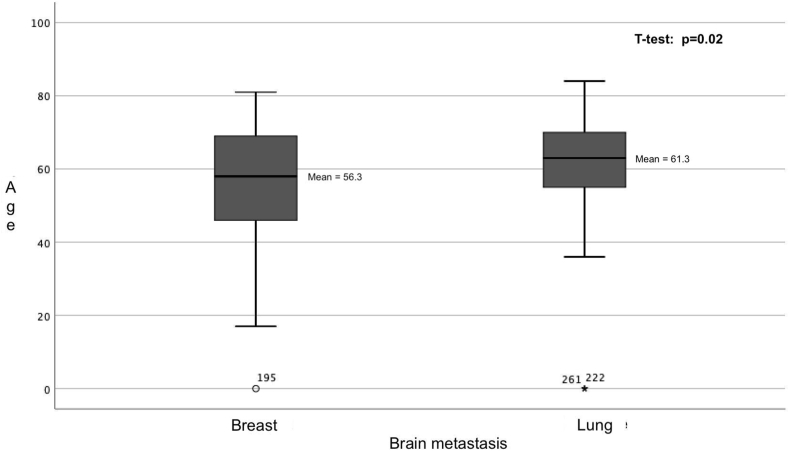
Box plot shows that patients with brain metastases from NSCLC have a later age at diagnosis than patients with breast cancer.

A a total of 314 patients affected by brain metastases from primitive lung cancer or breast cancer have been operated on in our Neurosurgical Units. The two analysis groups consisted of 179 BM patients from lung cancer (Group A) and 135 patients from breast cancer (Group B). Group A consists of 74 females (41.3 %) and 105 males (58.7 %), respectively; group B is composed of 132 females (97.7 %) and 3 males (2.2 %). There is a significant difference in the age of onset of BM symptoms between group A (mean 61.3 +- 13) and group B (mean 56.3 +- 14.7, p = 0.02, Fig. 1). Details are reported in Table 1.

**Table 1:** Patient population.

Brain metastases	Lung cancer BM	Breast BM	P-value
N° of patients (314)	179	135	
**Gender**			
- Female	74 (41.3 %)	132 (97.7 %)	
- Male	105 (58.7 %)	3 (2.2 %)	
**Age (Mean)**	61.3 ± 13	56.3 ± 14.7	**0.02**
**Clinical Debut**			
- Focal deficit	61 (34.3 %)	33 (24.3 %)	**0.05**
- Seizure	26 (14.5 %)	20 (14.8 %)	0.53
- Headache	39 (21.8 %)	30 (22.2 %)	1
- Incidental/follow-up	53 (29.7 %)	52 (38.5 %)	0.06
**Occurrence time**			**0.01**
- Synchronous	106 (59 %)	17 (12.6 %)	
- Metachronous	73 (40.8 %)	118 (87.4 %)	

### 3.1 Clinical group analysis

**Fig. 2:**
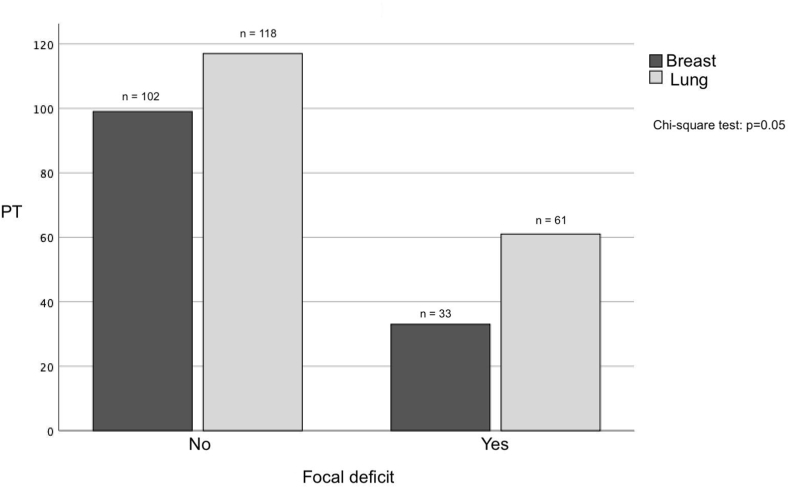
The bar chart shows that brain metastases from lung cancer most frequently debut with focal neurological disorders (PT = total number of patients).

**Fig. 3:**
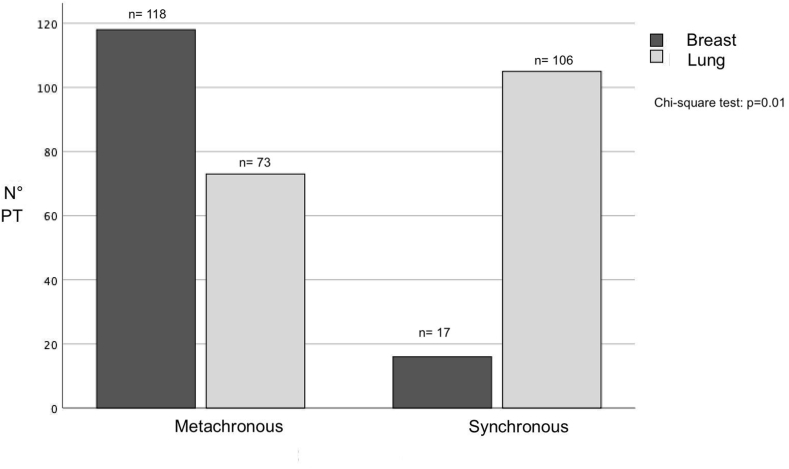
The bar chart shows that brain metastases from lung cancer most frequently have synchronous debut than breast cancer (PT = total number of patients).

Patients in group A most frequently clinically onset with focal symptoms or sensory-motor deficit (61 patients, 34.3 %, Fig. 2) with a significant difference from group B in which the most common onset was on follow-up imaging or incidentally (p = 0.05). This finding correlates with the type of metastasis onset: in fact, group B was more frequently radiologically diagnosed with metachronous BM beyond six months (118 patients, 87.4 %) from the primary tumor than group A, with a significant difference between the two groups (p = 0.01, Fig. 3). Details are reported in Table 2.

**Table 2:** The table shows the Radiological Comparison of BM cases from lung and breast.

**Brain metastases**	**Lung cancer BM**	**Breast BM**	**P-value**
**Side**			0.32
- Left	93 (52 %)	54 (40 %)	
- Right	84 (46.9 %)	79 (58.5 %)	
- Median	2 (1.11 %)	2 (1.48 %)	
**Lobe involvement**			0.55
- Frontal	51 (28.5 %)	42 (31.1 %)	
- Temporal	38 (21.2 %)	22 (16.3 %)	
- Parietal	32 (17.9 %)	28 (20.7 %)	
- Occipital	18 (10 %)	9 (6.7 %)	
**Subtentorial**	40 (22.3 %)	34 (25.1 %)	0.62
**Periventricular/deep seated**	29 (16.2 %)	26 (19.3 %)	1
**Volume (cm3)**	14.6	12.85	0.7
**Edema volume (cm3)**	54	48	1
**Multiple**	56 (31.3 %)	36 (27 %)	1
**Other metastases**	54 (30.2 %)	62 (46 %)	**0.05**
**Bone Involvement**	23 (12.8 %)	19 (14.1 %)	0.8

### 3.2 Radiological group analysis

Analyzing the MRI of all patients, no significant differences in localization are observed concerning the hemisphere, the most involved lobe, subtentorial lesions, and deep-seated/periventricular lesions between two groups.

The average volume of the lesions and perilesional edema were respectively 14.62 ± 18.5 cm3 and 54.21 ± 45.76 cm3 for group A and 12.85 ± 8.5 cm3 and 48.21 ± 35.76 cm3 for Group B without statistical significance between the two groups.

**Fig. 4:**
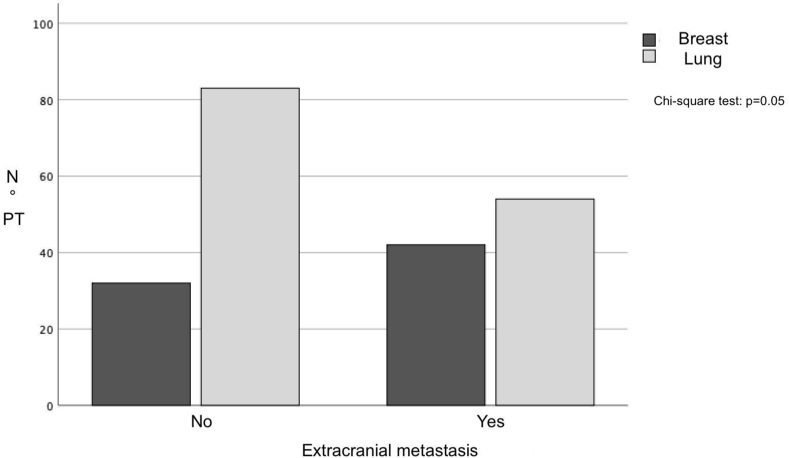
The bar chart shows that brain metastases from breast cancer most frequently have more extracranial metastases on debut than lung cancer (PT = total number of patients).

The presence of multiple intracranial lesions at initial radiological diagnosis was found in 56 patients in group A (31.3 %) and 36 patients (27 %) in group B, without finding a significant difference (p = 1). Interestingly, however, it was observed that following total-body re-staging CT scan performed immediately after the radiological diagnosis of BM, group B more frequently showed metastases in other extra-cranial sites (in 62 patients, 46 %, p = 0.05, Fig. 4), with a slight not-significant prevalence of bone metastatic lesions (14.1 % for group B versus 12.8 % for group A, p = 0.08). Details are reported in Table 3.

**Table 3:** The table shows the Comparison of surgical and outcome characteristics of BM cases from lung and breast.

Brain metastases	Lung cancer BM	Breast BM	P-value
**Surgical mass pattern**			0.01
- Solid	81 (45.3 %)	93 (68.8 %)	
- Cystic	90 (50.3 %)	39 (28.9 %)	
- Hemorragic	8 (5.9 %)	3 (2.2 %)	
**Surgical complication**	17 (9.5 %)	7 (5.2 %)	0.1
**Surgical resection plane**	154 (86 %)	117 (86.7 %)	1
**Performance status (KPS) - Mean**			
On debut	80	85	0.29
3 months	85	90	0.19
6 months	60	80	0.05

### 3.3 Surgical and outcome analysis

**Fig. 5:**
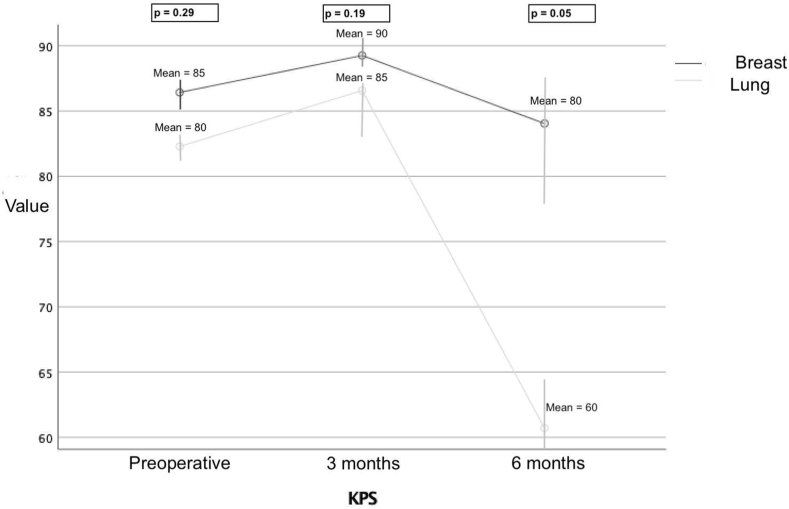
The graph shows that regarding the clinical outcome for the two groups, although no significant differences were found as regards the performance status at the time of diagnosis, and in the first 3 months of surgery, a significant difference was found in the drop in performance for as regards group A.

**Fig. 6:**
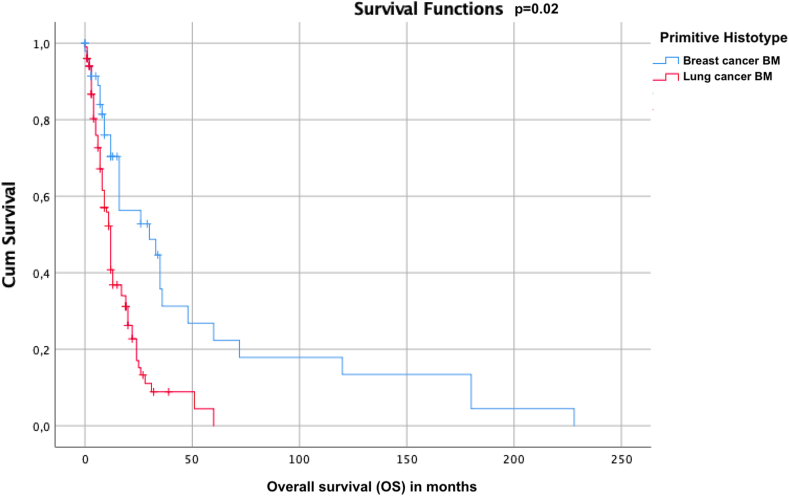
Kaplan–Meier graph shows a significant difference in survival between Lung cancer BM (mean overall survival, OS = 15.9) and breast cancer BM (mean OS = 52.1).

From the point of view of the surgical pattern of density and appearance, breast cancer metastases were more frequently solid than those from lung cancer (68.8 % versus 45.3 %, respectively, p = 0.01). The BMs of group A appeared more varied in morphology than group B's. From the point of view of adherence to the surrounding tissue of the tumor mass, no significant differences were found between the two groups in which an en-bloc resection of the tumor was obtained respectively in 86 % of the cases of group A and in 86.7 % of the cases of group B. Considering post-operative surgical complications there is a slight prevalence not-significant of re-bleeding of the cord in group A (9.5 %) compared to group B (5.2 %, p = 0.1), no other forms of early complications such as infections or CSF fistulas were found for the two groups. Regarding the clinical outcome for the two groups, no significant differences were found as regards the performance status at the time of diagnosis, and in the first 3 months of surgery, while a significant difference was found in the drop in performance for as regards group A (Fig. 5). Mean survival from diagnosis of brain metastasis appears to be significantly different between group A and group B (Mean = 15.9, sd = 1.76, CI-95 % = 12.47–18.37 for Lung cancer BM versus Mean = 52.1, sd = 12.5, CI-95 % = 27.5-76-7 for Breast cancer BM, Breslow–Wilkinson test p = 0.02, Fig. 6). No further analyses were performed at this point as the differences were evident according to the different biological and molecular types of tumors and the different aggressiveness of the primary tumor.

## 4 Discussion

**Fig. 7:**
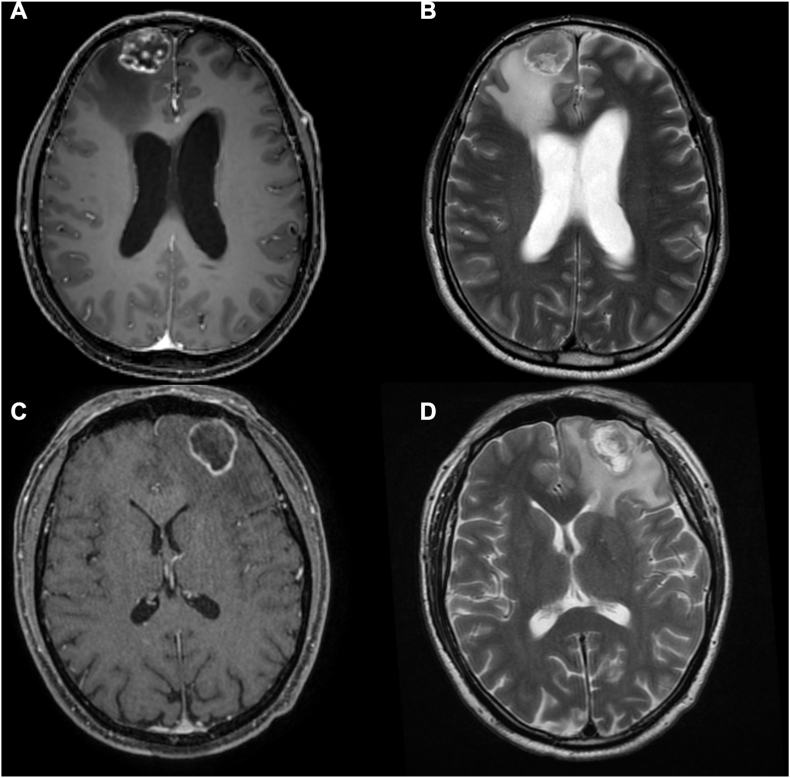
The image shows MRI images of two patients with frontal brain metastasis. In case A, the breast cancer patient has a less edemigenous solid metastasis than the patient in case B with NSCLC, who has a cystic mass with diffuse edema.

BMs from lung and breast cancer are the most frequent types of secondary intracranial lesions in adults since melanoma patients were nowadays excluded as they have seen significant progress in the development of systemic therapies to improve control of the primary disease, reducing the percentage who develop BM.^20^, ^21^, ^22^, ^23^ Biologically and prognostically, the two types cannot be comparable since they are two different clinical entities, but at the time of acute clinical onset where there is radiological evidence of a surgically treatable contrast capturing lesion their appearance can be very similar and difficult to distinguish to other tumors (Fig. 7).^23^, ^24^, ^25^, ^26^ In this study, the natural histories of breast and lung cancer patients are consistent with those reported by others.^27^^,^^28^ Still, under clinical, radiological and surgical aspects, there are some interesting differences especially regarding age, clinical debut, timing, extracranial metastases and surgical features. The main features are reassumed in Table 4.

**Table 4:** The table summarizes the main differences, identified by the present study, in clinical, radiological, and surgical characteristics between the two tumor histotypes.

Brain metastases main features		
	Lung metastases	Breast metastases
Mean Age	>60 years old	<60 years old
Onset	Synchronous	Metachronous
Diagnosis	With focal neurological deficit	During follow-up
Pattern	Cystic	Solid
Surgical features	Single brain metastases	Extracranial metastases
		Bone involvement
Performance status (KPS)	KPS = 60 at 6 months	KPS = 80 at 6-months
Overal survival	16 +- 1.75	52 +- 12.5

### 4.1 Clinical features

The analysis shows a difference in the age of onset of BM in the two histological types: according to the literature^28^ patients with BMs from lung carcinoma are older compared with those with metastases from other anatomical sites, with a mean age of 58 years; in metastases from breast carcinoma, the mean age is about 49 years, therefore, younger than in other localizations.^28^

We also confirm that BMs from non-small cell lung cancer (NSCLC) are more often synchronous (59 %) than metachronous (40.8 %); in contrast, breast metastases occur more often after the primary diagnosis (87.4 % metachronous vs 12.6 % synchronous).^29^ The time of synchronous or metachronous presentation could impact prognosis and thus decisions between conservative or surgical treatment.^30^ This relates to some studies done in NSCLC on prognosis, which is more unfavorable in patients with synchronous metastases, who have a median survival of 2.9 months, while metachronous metastases have a median survival of 3.4 months.^31^ The average interval between initial cancer diagnosis and identification of BM is short for lung cancer with a range of 2–9 months, with >50 % of patients with lung cancer being diagnosed with a BM within the first year after initial diagnosis.^21^^,^^32^

Almost all received adjuvant chemo- or endocrine therapy of proven efficacy. Relapse, when it occurred, was delayed for years and was usually in the lungs and bones; such metastases often respond to systemic therapies. Brain involvement usually became clinically detectable only after other distant metastases had been found or during the follow-up.^33^

Our study shows that a clinical debut with neurological impairment permits an earlier diagnosis of BM in lung cancer than in breast cancer, so a special focus was given to the clinical onset. BM may present with headache, nausea, and vomiting, symptoms more common in breast cancer metastases.^34^ 20–40 % of patients initially present with focal neurological symptoms,^35^ with greater prevalence seen in lung cancer metastases.

The incidence of epilepsy varies from 20 to 35 %,^36^ with a higher frequency for lung primary metastases (29 %)^37^ and 26.4 % for breast metastases.^38^ About 10 % of patients with BM who clinically debut with seizures develop epilepsy during the disease.^39^ The precise mechanism by which metastases can cause epilepsy both pre- and post-operatively is still unclear, considering that metastases do not have an infiltrative character, unlike primary lesions.^40^ We found that 14.5 % of patients with metastases from lung carcinoma and 14.8 % of patients with metastases from breast carcinoma began with seizures, consistent with data reported in the literature^41^ and with no apparent correlation with biological type. In contrast, there seems to be a correlation between age, location, and headache: young patients with subtentorial metastases, with larger masses, and who present with headaches have a lower risk of developing seizures.

### 4.2 Radiological features

We compared the MRI scans of all patients in this series since data on BMs' location, morphology, and macroscopic structure can be obtained with conventional MRI sequences. Most BMs occur in the cerebrum (around 80 %),^42^ followed by the cerebellum and brainstem,^43^ with an overall distribution of metastases in correspondence of the cerebral gray–white junction,^5^ primarily in the territory of the middle cerebral artery (MCA) and posterior cerebral artery (PCA).^44^, ^45^, ^46^, ^47^

In our analysis, contrary to what is often reported in many studies,^5^^,^^48^, ^49^, ^50^ there is no prevalence of specific localization on a brain lobe between the two tumor types. Our data also confirm a not-significant higher incidence of cerebellar metastases in breast cancer patients than in lung cancer patients without any correlations between histotype and localization.

Considering the presence of multiple lesions, although a slight prevalence of multifocality in breast carcinoma metastases is reported in the literature,^51^, ^52^, ^53^ no significant difference between the two types in the presence of multiple metastases was found in our study. Some authors reported that is possible that the presence of multiple metastases is correlated with the longer survival of breast cancer patients compared with those with lung cancer.^52^^,^^54^ These data, however, are contradictory to the various published case collections.^55^

It is reported that the median survival of breast cancer patients with BM varies according to HER-2 subtype with the triple-negative forms having worse prognosis (about 6 months), than HER 2 positive HR (about 21 months).^56^ In patients with metastasis from lung carcinoma, seems that the EGFR expression correlates with prognosis,^57^ although our recent study^23^ on this collection did not confirm this finding. Patients with metastasis from breast carcinoma present with more advanced intracranial disease than patients with metastasis from NSCLC but after treatment, no differences are reported between the two groups.^53^ Further, we showed that even after treatment, distal performance status was measured as KPS precipitates in patients with lung cancer.

Another relevant finding is the greater presence of extracerebral metastasis in breast carcinoma patients^58^ with greater prediction for bone metastasis. Indeed, bone marrow is a specific organ of research for breast metastatic cells. Patients with metastasized breast cancer have a high susceptibility to bone, liver, and lung.^59^ The brain is the most common and often the only site of extra-thoracic metastases from lung cancer. In light of the above review and study, we can confirm that breast carcinoma is not more aggressive than NSCLC but is diagnosed later and in an advanced progression.

### 4.3 Surgical features

Surgery represents the treatment of choice in cases of significant mass effect and when debulking is necessary for immediate symptom relief and/or improvement of quality of life: if a patient has a single lesion that is small but symptomatic or has extensive perilesional edema and/or creates seizures refractory to medical treatment, he or she may be a candidate for surgery.^60^ Therefore, the surgical aspect of BM is essential for patient safety, outcomes, and diagnostic guidance. It has been suggested that BMs from breast cancer may also be constitutively less aggressive than those from lung cancer, since it has been reported that brain edema is greater in BMs from lung cancer (partially confirmed in our analysis); similar to primary lung cancers, their metastases may also elicit greater inflammation than breast cancers.^52^

The mean volume of breast cancer metastases appears to be smaller than that of lung metastases, which is partially consistent with the size reported in the literature.^60^

The gross appearance of tumor mass is largely non-specific apart from its tendency to have sharp borders in contrast to primary brain tumors, most of which typically have infiltrating borders. Softening of the surrounding brain parenchyma due to edema is often prominent and sometimes disproportional to the size of the lesions.^6^

During the surgical resection BMs can appear with different consistencies. Tumor masses usually appear solid, while cystic forms are due to central necrosis or intratumoral hemorrhage.^61^ Ebinu et al showed that cystic lesions are in 51 % of lung carcinomas and 10 % from breast.^62^ In contrast, other studies have reported a greater presence of cystic lesions in metastases from breast carcinoma followed by lung.^63^^,^^64^ Our reported data confirm those reported by Ebinu with a prevalence of cystic lesions in metastases from lung carcinoma.

When possible, total surgical resection (GTR) is the goal of surgery, as it improves patient outcome.^65^ Removal can be in “piecemeal (internal debulking followed by removal of the capsule in multiple pieces) or “en bloc” (circumferential dissection along tumor–brain interface without violation of the tumor capsule). When possible, the treatment most commonly performed in our institution was en bloc resection. For this reason, our findings about the “surgical pattern” in the meaning of consistency of the tumor mass is necessary and valuable it. Nevertheless, GTR in our study was reported in 86 % of cases, whereas data in the literature show a rate of 61.5 % by Kamp^55^ and 62.4 % by Junger.^6^^,^^65^

### 4.4 Limitations and further study

The study reported has several limitations. The most important concern the fact that analyzing groups of patients with a deeply different pathology, such as lung and breast cancer, may lead to scientifically incorrect conclusions. For this reason the study focuses on the differences and the identification of recognition predictive factors useful to the surgeon in the initial phase of the treatment process especially when the initial diagnosis is not known and does not further explore the differences in prognosis between the two groups. The second important limitation is given by the fact that the two groups analyzed are patients affected by BM with a performance status, age and number of lesions that are compatible with a neurosurgical evaluation for treatment and diagnosis, thus excluding the more aggressive and intractable. We deliberately chose not to consider the third most common group of BMs, namely those from metastatic melanoma, due to some peculiar clinical, radiological and prognostic differences already known in the literature; moreover, from our retrospective analysis, the number of such patients was very small (<30 patients) thanks above all to the improvement of adjuvant therapies applied in recent years to melanoma.

## 5 Conclusions

The objective of this research was to examine clinical, radiological, and surgical factors in patients with BMs resulting from lung and breast cancers who were treated surgically. The analysis of surgical data indicates that BMs from breast carcinoma tend to occur in younger patients in a metachronous manner, with a preference for infratentorial localizations, often in patients with other extracranial localizations, particularly at the bone level. In contrast, BMs from lung cancer tend to be synchronous, more often cystic, and with greater edema volume, reflecting a more aggressive behavior of lung carcinoma. The data presented in this study suggest that while the two most common forms of BM exhibit distinctions with respect to clinical onset, age, tumor location, presence of extra-cranial metastases, and lesion morphology from a strictly surgical standpoint, they are indistinguishable with regard to outcome, demonstrating comparable resection rates and a low risk of complications.

## CRediT authorship contribution statement

**Daniele Armocida:** Writing – review & editing, Methodology, Conceptualization. **Giuseppa Zancana:** Writing – original draft, Data curation. **Andrea Bianconi:** Investigation, Data curation. **Fabio Cofano:** Methodology, Investigation, Formal analysis. **Alessandro Pesce:** Validation, Software, Formal analysis. **Brandon Matteo Ascenzi:** Supervision. **Paola Bini:** Data curation. **Enrico Marchioni:** Data curation. **Diego Garbossa:** Visualization, Validation, Supervision. **Alessandro Frati:** Visualization, Supervision, Conceptualization.

## Declaration of competing interest

We wish to draw the attention of the Editor to the following facts which may be considered as potential conflicts of interest and to significant financial contributions to this work.

We wish to confirm that there are no known conflicts of interest associated with this publication and there has been no significant financial support for this work that could have influenced its outcome.

We confirm that the manuscript has been read and approved by all named authors and that there are no other persons who satisfied the criteria for authorship but are not listed. We further confirm that the order of authors listed in the manuscript has been approved by all of us.

We confirm that we have given due consideration to the protection of intellectual property associated with this work and that there are no impediments to publication, including the timing of publication, with respect to intellectual property. In so doing we confirm that we have followed the regulations of our institutions concerning intellectual property.

We further confirm that any aspect of the work covered in this manuscript that has involved either experimental animals or human patients has been conducted with the ethical approval of all relevant bodies and that such approvals are acknowledged within the manuscript.

We understand that the Corresponding Author is the sole contact for the Editorial process (including Editorial Manager and direct communications with the office). He/she is responsible for communicating with the other authors about progress, submissions of revisions and final approval of proofs. We confirm that we have provided a current, correct email address which is accessible by the Corresponding Author and which has been configured to accept email from.
